# Cyclophosphamide Exposure Causes Long-Term Detrimental Effect of Oocytes Developmental Competence Through Affecting the Epigenetic Modification and Maternal Factors’ Transcription During Oocyte Growth

**DOI:** 10.3389/fcell.2021.682060

**Published:** 2021-06-07

**Authors:** Weijie Yang, Yerong Ma, Jiamin Jin, Peipei Ren, Hanjing Zhou, Shiqian Xu, Yingyi Zhang, Zhanhong Hu, Yan Rong, Yongdong Dai, Yinli Zhang, Songying Zhang

**Affiliations:** ^1^Assisted Reproduction Unit, Department of Obstetrics and Gynecology, Sir Run Run Shaw Hospital, Zhejiang University School of Medicine, Hangzhou, China; ^2^Key Laboratory of Reproductive Dysfunction Management of Zhejiang Province, Department of Obstetrics and Gynecology, Hangzhou, China

**Keywords:** cyclophosphamide, maternal factor, histone modification, methylation, primordial follicle, oocyte, embryo

## Abstract

Cyclophosphamide (CTX) is widely used in various cancer therapies and in immunosuppression, and patients can still have babies after CTX chemotherapy. CTX directly causes primordial follicle loss with overactivation and DNA damage-induced apoptosis. Previous studies have shown that maternal exposure to CTX before conception increases the incidence of birth abnormalities and alters the methylation of genes in the oocytes of offspring. Mice were treated with a single dose of CTX (100 mg/kg) at post-natal day 21 and sacrificed 47 days later when primordial follicles surviving chemotherapy developed to the antral stage. Acute DNA damage and acceleration of the activation of primordial follicles after CTX treatment were repaired within several days, but the remaining follicle numbers remarkably decrease. Although partial surviving primordial follicle were developed to mature oocyte, oocyte quality hemostasis was impaired exhibiting aberrant meiosis progression, abnormal spindle and aneuploidy, mitochondrial dysfunction and increased endoplasmic reticulum stress. Thereafter, embryo development competency significantly decreased with fewer blastocyst formation after CTX exposure. CTX treatment resulted in alteration of DNA methylations and histone modifications in fully grown GV oocytes. Single-cell RNA-seq revealed CTX treatment suppressed multiple maternal genes’ transcription including many methyltransferases and maternal factor YAP1, which probably accounts for low quality of CTX-repaired oocyte. *In vitro* addition of lysophosphatidic acid (LPA) to embryo culture media to promote YAP1 nuclear localization improved CTX-repaired embryo developmental competence. This study provides evidence for the consistent toxic effect of CTX exposure during follicle development, and provide a new mechanism and new insights into future clinical interventions for fertility preservation.

## Introduction

With advancements in diagnosis and treatment, the survival rate of patients with childhood malignant cancer has increased. Nowadays, more than 80% of child cancer patients will survive past 5 years (Bertoldo et al., 2020), but the long-term effects of cancer treatment are still unclear. Cancer treatment may affect ovarian function and oocyte competency (Winship et al., 2018; Spears et al., 2019). Radiation and chemotherapy agents directly damage ovarian follicles, stromal cells and the composition of the extracellular matrix in the ovary (Ben-Aharon and Shalgi, 2012).

Cyclophosphamide (CTX) is one of the most prevalent alkylating chemotherapy drugs and is widely used in the treatment of breast cancer, lymphoma, leukemia and various of childhood and adult malignant tumors (Cronin et al., 2018). CTX is also used in a range of autoimmune disease therapies and prior immunosuppression of stem cell transplantation (Leroy et al., 2015; Freeman et al., 2016). Many studies have focused on the acute toxic injury of CTX on ovarian function. CTX can directly cause primordial follicle loss with DNA damage-induced apoptosis and overactivation. Commonly, exposure to CTX is not cell cycle dependent; it induces γ-H2AX-positive DNA double strand breaks (DSBs), alters the regulation of pro-apoptotic genes and induces primordial follicle death in the ATM- and PARP1-dependent DNA repair pathways (Morgan et al., 2012; Ganesan and Keating, 2016). In addition, CTX stimulates the PI3K-PTEN-AKT pathway in parallel, which disrupts the balance regulation of dormant primordial follicles and causes follicle reserve loss. Phosphoramide mustard, one of the metabolites of CTX, was reported to affect mitochondrial function, leading to decreased transmembrane potential and the accumulation of cytosolic cytochrome C (Spears et al., 2019). Moreover, CTX induces the overproduction of reactive oxygen species (ROS) mediated by another metabolite, acrolein. *In vivo* animal models show a disruption in lipid peroxidation and reduced superoxide dismutase activity, which eventually damage ovarian function (Detti et al., 2013; Saleh and Mansour, 2016).

In mammals, it is widely suggested that oocytes cannot be renewed during reproductive life (Skinner, 2005). Damage to the follicle population is closely related to infertility, ovarian function failure, and even premature ovarian insufficiency (POI) (Zhang et al., 2019). A previous study suggested that female cancer survivors treated with chemotherapy still have the ability to become pregnant and deliver (Chow et al., 2016). CTX exposure is associated with a decreased likelihood of pregnancy and live birth rate. Previous research on clinical cohorts revealed that maternal exposure to CTX before conception will increase the incidence of a birth abnormality and resulted in delayed growth of the next generation with alteration of methylation of three imprinted genes (*H19, Igf2r*, and *Peg3*) in the offspring’s oocytes (Meirow et al., 2001; Di Emidio et al., 2019). Therefore, a proportion of primordial follicles can repair DNA damage and eventually escape CTX insult. The long-term side effects of CTX and potential role of CTX in oocyte survival are still unknown.

In mice, there are two classes of primordial follicles with distinct and age-dependent developmental paths: the first wave of primordial follicles with simultaneous activation after birth and the primordial follicles with gradually activation in adulthood (Zheng et al., 2014). The second wave of primordial follicles that reach the antral stage require approximately 47 days in a mouse model. Mature oocyte quality alteration is associated with spindle morphology, chromosome aneuploidy and subcellular organelles distribution, such as mitochondria and the endoplasmic reticulum (Li et al., 2020). Oocyte quality is closely coupled with the follicle and oocyte growth states, which determines the subsequent competency of maturation, fertilization, and embryonic development (Dou et al., 2017). During follicle growth, massive maternal mRNAs are transcribed and stored in immature GV oocytes, and meanwhile DNA methylations and histone methylations are established in fully grown GV oocytes. During oocyte meiotic maturation, fertilization and early embryo development, substantial maternal mRNAs are translationally activated followed by gradually degradation (Sha et al., 2019). Many studies have demonstrated that oocyte-inherited maternal factors govern the early embryo developmental fate, such as *Yap1*, *Dnmt1*, *Btg4*, and *Stella* (Yu et al., 2016a, b; Li et al., 2018). Since some remaining follicles can survived from CTX exposure, whether molecular changes occurs in these surviving oocytes is unclear.

YAP1 is a transcriptional co-activator of TEAD family transcription factors in Hippo signaling pathway (Chen et al., 2010). Maternal YAP1 inherited from oocytes is essential for zygotic genome activation (ZGA) as maternal knockout of *Yap1* impedes early embryo development (Yu et al., 2016a). LPA can be found in many animal biological fluids and enriched in follicular fluid (Sheng et al., 2015), which previously demonstrated that LPA can activate YAP1 and inhibit the Hippo signaling pathway both in culture cell lines and embryo culture systems (Sha et al., 2020). A recent study showed that the addition of LPA to embryo culture medium promoted YAP1 nuclear localization and improved the blastocyst formation rates of embryos (Yu et al., 2016a). Whether LPA improves CTX-damaged embryo development is need to be investigated.

In this study, we aimed to determine whether short exposure to CTX would have a long-term effect on oocytes surviving from primordial follicles during therapy. We firstly demonstrated CTX causes mitochondrial dysfunction and ER stress, which impacts oocyte quality and embryo developmental potential. Furthermore, we found both DNA methylation and histone methylation were altered. The single-cell RNA sequencing (RNA-seq) results revealed CTX insult suppressed multiple maternal transcripts accumulation, especially relative to chromatin organization. Finally, addition of LPA into embryo culture medium improves embryo development derived from CTX-damaged oocytes. Our study provides important evidence of the long-term detrimental effect of CTX on surviving primordial follicles and indicate a potential underlying mechanism.

## Materials and Methods

### Experimental Animal Model

Female ICR mice were obtained from the Animal Center of Sir Run Run Shaw Hospital and maintained in a standard temperature-controlled environment under a 12-h light/dark cycle. The experiments were performed with the approval of the Committee on the Ethics of Animal Experiments at Zhejiang University. Three-week-old ICR female mice were injected intraperitoneally with a single dose of normal saline (N.S.) or 100 mg/kg CTX (Baxter, Italy). The ovaries were collected after treatment for 1, 3, 7, 21, and 47 days for further detection. After 47 days of injection, the primordial follicles that were damaged from CTX chemotherapy developed into the antral stage. The oocytes were retrieved and superovulated from surviving mice for the following experiment.

### Oocyte Collection and Culture

Mice with or without CTX treatment for 47 days were injected with 5 IU of pregnant mare serum gonadotropin (PMSG; Ningbo Sansheng Pharmaceutical, China) and humanely euthanized 46 h later. Oocytes at the GV stage were harvested and cultured in M2 medium (M7167; Sigma-Aldrich) under mineral oil (M5310; Sigma-Aldrich) at 37°C in 5% CO_2_.

### *In vitro* Fertilization and Embryo Culture

After injection with NS or CTX for 47 days, female mice were intraperitoneally injected with 5 IU of PMSG followed by human chorionic hormone (hCG; Ningbo Sansheng Pharmaceutical, China) 46 h later. Sperm were collected from 8-week_–_old male mice into HTF media (M1130, Nanjing Aibei Biotechnology, China) and incubated under oil for 1 h at 37°C in 5% CO_2_ for capacitation. MII stage oocytes were harvested from the oviducts 16 h after hCG injection and placed into HTF media with sperm for 6 h. After fertilization, zygotes were removed and placed into small droplets of KSOM media (M1130, Nanjing Aibei Biotechnology, China). Embryonic development was monitored and assessed from the 2-cell to blastocyst stages.

### Single-Cell RNA-Seq

GV-stage fully grown oocytes without zona pellucida were collected and lysed with 4 μL of lysis buffer (0.2% Triton X-100, RNase inhibitor, 10 mM dNTP mix, and 10 μM oligo-dT primer). Each sample contained one oocyte, and 0.1 μL of ERCC spike-in RNA (Life Technologies, 4456740) was added as an external control. cDNA synthesis and amplification were performed following the Smart-seq2 protocol (Picelli et al., 2014). Sequencing libraries were constructed by KAPA KK8514 (Roche) according to the instructions. The library was sent to Annoroad Gene Technology Corporation (Beijing, China) and sequenced on an Illumina HiSeq X Ten for 150-bp paired-end sequencing with at least 4 million reads per sample. During analysis, all the generated raw reads were filtered to obtain clean reads in FASTQ format. The reads were mapped to the mouse reference genome (mm10) using TopHat (version 2.0.9). Sequencing reads mapping to ERCC “spike-ins” were used to estimate technical “noise” levels (Brennecke et al., 2013). Differential gene expression between control and CTX-treated oocytes and two clusters was determined by Cufflinks (version 2.1.1) and R (version 3.5.1). Cutoff threshold for significance is log2 (foldchange) ≥ 1 and Multi-test adjusted *p* ≤ 0.05. Gene Ontology (GO) and Kyoto Encyclopedia of Genes and Genomes (KEGG) pathway analyses were performed using the clusterProfiler (version 3.18.1). Processed RNA-seq data are presented in Supplementary Tables 1, 2 and the single cell quality control data is shown in Supplementary Table 3.

### Histological Analysis and Follicle Counting

Ovaries were collected and fixed in 10% buffered formalin, embedded in paraffin and cut into serial sections (5 μm). The sections were then stained with hematoxylin and eosin. To evaluate follicular development in mice with or without CTX treatment, all follicles with a visible nucleus were counted every fifth section as previously described (Yang et al., 2020). In brief, primordial follicles were surrounded by a flat layer of granulosa cells, while primary follicles contained a layer of cuboidal granulosa cells.

Multiple layers of granulosa cells surrounding follicles were determined to be in the secondary stage, and follicles with a visible cavity between oocytes and granulosa cells were considered to be in the antral stage. Atretic follicles contained degenerating oocytes, disorganized granulosa cells or apoptotic bodies (Sun et al., 2015). All sections were counted by two independent individuals for comparison.

### Immunoblot Analysis

Proteins were extracted with RIPA lysis buffer (R0010, Solarbio, China) containing a protease inhibitor cocktail (P8340, Sigma-Aldrich, United States). Proteins were separated by electrophoresis, and after electronic transfer, the membranes were blocked for at least 1 h and then incubated overnight at 4°C with specific antibodies. Primary antibodies used in the experiments were as follows: β-actin (66009,1:3,000, Proteintech, China), p-mTOR (Ser2448) (5536, 1:1,000, CST, United States), mTOR (2983, 1:1,000, CST, United States), p-AKT (Ser473) (4060, 1:1,000, CST, United States), AKT (4691, 1:1,000, CST, United States), p-rpS6 (Ser240/244) (5364, 1:1,000, CST, United States), rpS6 (2217, 1:1,000, CST, United States), γ-H2A.X (Ser139) (9718, 1:400, CST, United States), cleaved PARP (9548, 1:1,000, CST, United States) and PARP (9532, 1:1,000, CST, United States). After washing with TBST buffer, the membranes were incubated with HRP-conjugated goat anti-rabbit or mouse IgG secondary antibodies (7074,7076, 1:3,000; CST, United States) and visualized by enhanced chemiluminescence (WBKLS0500, Millipore, United States) to detect proteins.

### Real-Time Quantitative PCR

Five oocytes were collected and lysed in lysis buffer according to the single-cell RNA-seq protocol described above. Samples were subjected to reverse transcription and amplification by PCR for 10 cycles. The PCR products were purified and diluted for real-time qPCR templates. The procedure was performed using SYBR Green Mix (Vazyme, China) on a CFX-Connect platform (Bio-Rad, United States), and all the primer sequences used are listed in Supplementary Table 4. The specificity of the PCR products was assessed by melting curve analyses, and amplicon size was determined by electrophoresis in 2% agarose gels. The relative transcript levels of samples were compared with those of the control, and the fold changes are shown.

### Immunofluorescence

Ovarian samples were fixed in 4% paraformaldehyde overnight and dehydrated with a series of 10–30% sucrose solutions. The frozen sections were permeabilized with 0.3% Triton X-100 (T8787, Sigma-Aldrich, United States) for 15 min at room temperature. GV and MII oocytes were collected and fixed in 3.7% paraformaldehyde for 30 min and then permeabilized with 0.5% Triton X-100 for 20 min at room temperature. For 5-mC and 5-hmC staining, permeabilized oocytes were incubated in 4 N HCl solution for 10 min, followed by neutralization in Tris-Cl, pH 8.0, for another 10 min. After blocking in 1% BSA solution for 1 h, oocytes or sections were incubated with primary antibodies overnight at 4°C. The primary antibodies used were as follows: γ-H2A.X (Ser139) (9718, 1:400, CST, United States), YAP (14074, 1:100, CST, United States), FITC-α-tubulin (F2168, 1:200, Sigma-Aldrich, United States), beta-tubulin (ab18207, 1:200, Abcam, United States), GRP78 (ab21685, 1:200, Abcam, United States), H3K4me3 (Ab1012, 1:400, Abcam, United States), H3K9me3 (Ab8898, 1:400, Abcam, United States), 5mC (Ab10805, 1:400, Abcam, United States), and 5-hmC (39769, 1:400, Active Motif, United States). After washing in 1% BSA/PBS, oocytes were incubated with an Alexa Fluor 488- or 568-conjugated goat secondary antibody (A32731, A32723, A-11011, A-11004, 1:400, Invitrogen, United States) for 45 min at room temperature. The nuclei were then stained with Hoechst 33342 (H3570, 1:1,000, Invitrogen, United States) for 15 min. For MitoTracker and ER-Tracker staining, live oocytes were incubated with MitoTracker Red CMXRos (M7512, 1:1,000, Invitrogen, United States) or ER-Tracker Red (E34250, 1:1,000, Invitrogen, United States) for 1 h. All the oocytes and slides were observed by confocal microscopy (Zeiss, LSM800, Germany).

### Chromosome Spreading

Zona pellucida-free oocytes were fixed in a solution containing 1% paraformaldehyde, 0.15% Triton X-100, and 3 mM DTT (Sigma-Aldrich) on glass slides and dried in air. The glass slides were washed with PBS, and immunofluorescent staining was performed as described above. The number and pairs of chromosomes were observed by confocal microscopy.

### Measurement of JC-1, ROS, and ATP

To determine the mitochondrial membrane potential and ROS content in oocytes, the procedures were performed following the instructions of the JC-1 Mitochondrial Membrane Potential Assay Kit (C2006, Beyotime, China) and the ROS Kit (S0033S, Beyotime, China). In brief, MII oocytes were incubated in M2 medium with 2 μM JC-1 probe or 10 μM DCFH-DA at 37°C in the dark for 30 min. Next, oocytes were washed to remove excess staining solution. All the live oocytes were observed, and the relative fluorescence intensity was calculated by confocal microscopy. For ATP content measurement, a single oocyte without zona pellucida was collected into a tube and lysed. The procedure was conducted according to the instructions of the ATP Assay Kit (S0026, Beyotime, China).

### Statistical Analysis

All measurements were performed independently at least in triplicate. GraphPad 8.0 and SPSS 23.0 software were used for all statistical analyses. All quantitative data are shown as the means ± SEMs. Difference between two groups were evaluated by an unpaired t test. For more than two groups, one-way ANOVA was used to evaluate differences. *P* < 0.05 was considered to indicate statistical significance.

## Results

### Short Exposure of CTX Decreases Follicle Numbers

**FIGURE 1:**
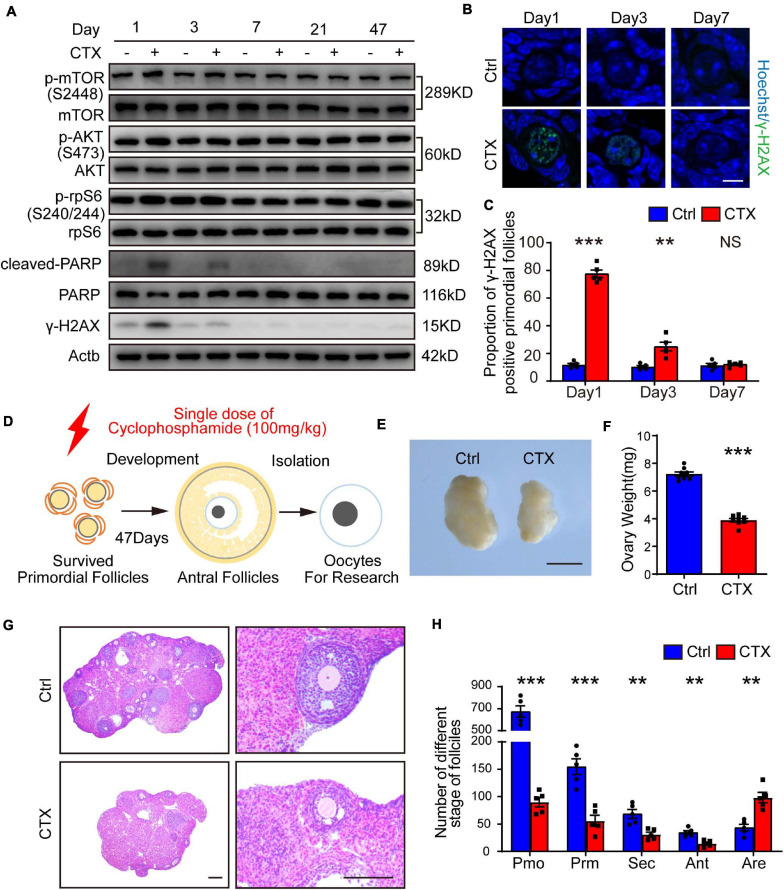
CTX treatment caused activation and acute DNA damage of primordial follicles and impaired long-term follicle development. **(A)** Western blot analysis of dynamic alterations in the PI3K-AKT-mTOR signaling pathway, DNA damage marker γ-H2AX and apoptotic marker PARP. Ovary samples were collected after injection of CTX on days 1, 3, 7, 21, and 47. **(B)** Immunostaining of γ-H2AX in residual primordial follicles after CTX exposure. Scale bar = 10 μm. **(C)** Statistical analysis of the proportion of γ-H2AX-positive primordial follicles in the residual follicle pool. **(D)** Graphical abstract of experiment design and time of oocyte collecting. **(E)** Morphology of ovaries collected after 47 days of exposure to CTX. Scale bar = 500 μm. **(F)** Ovary weight decreased after 47 days of CTX treatment. **(G)** Histological analysis of ovary morphology and residual primordial follicles in the cortex. Scale bar = 100 μm. **(H)** Statistical analysis of different stages of follicles with or without CTX treatment. Pmo, primordial follicle; Prm. primary follicle; Sec, secondary follicle; Ant, antral follicle; Are, atretic follicle. Data was presented as mean ± SEM. NS, no significant, **P < 0.01, ***P < 0.001.

Previous research showed that primordial follicles from pre-pubertal mice reached the antral stage after approximately 47 days (Figure 1D; Zheng et al., 2014). We first detected continuous dynamic alterations in the PI3K-AKT-mTOR pathway and DNA damage effects after a single injection of CTX (100 mg/kg). As shown in Figure 1A, the phosphorylation of AKT (Ser473) and mTOR (Ser2448) were upregulated within 1 day and decreased from 3 days after treatment; these findings were consistent with the changes in the DNA damage marker γ-H2AX and the cleavage of poly (ADP-ribose) polymerase (PARP), which indicates apoptotic levels (Figures 1B,C). From 7 day and behind, activation of the signaling pathway and the detrimental effects returned to baseline levels. We also measured DNA damage in primordial follicles by immunostaining for γ-H2AX and found a significant increase after 1 day (11.79 ± 1.03% vs. 77.54 ± 2.78%, *n* = 5) of CTX exposure and a decline after 3 days (10.41 ± 0.91% vs. 24.99 ± 3.16%, *n* = 5). There was no comparable difference in DSBs between preserved primordial follicles on day 7 (11.45 ± 1.37% vs. 12.10 ± 0.78%, *n* = 5) (Figures 1B,C). Regarding the detrimental effect on ovarian and follicle development, we observed that the ovarian volume of the CTX-insulted group was smaller than that of the control group (Figure 1E), and the weight was decreased markedly (7.23 ± 0.16 vs. 3.89 ± 0.14 mg, *n* = 8) (Figures 1E,F). Histological analysis revealed impaired follicle development in CTX-treated ovaries (Figure 1G). We counted follicles and found that the numbers of both non-growing and growing follicles after CTX exposure were reduced significantly, while the number of atretic follicles was increased (primordial 674.60 ± 51.24 vs. 90.20 ± 8.68, primary 154.6 ± 14.48 vs. 56.00 ± 10.11, secondary 68.60 ± 8.04 vs. 31.00 ± 4.30, antral 34.60 ± 3.41 vs. 14.60 ± 2.56, atretic, 43.40 ± 5.98 vs. 98.40 ± 9.31, *n* = 5) (Figure 1H). These results suggest overactivation and acute DNA damage in oocytes that can be repaired in surviving primordial follicles but long-term adverse effects on ovarian and follicle development.

### CTX Impairs Oocyte Maturation and Causes Defects in Ovulated Oocytes

**FIGURE 2:**
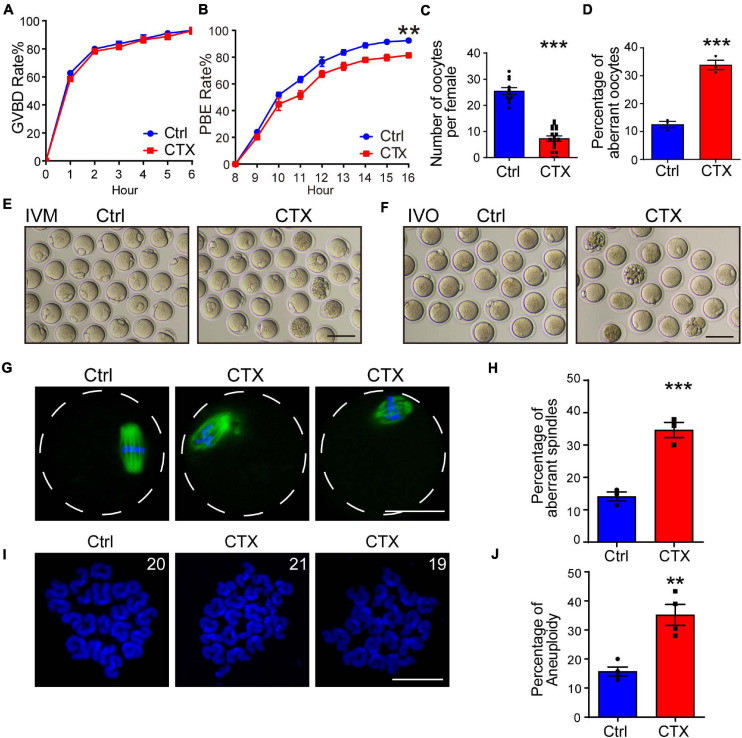
Effects of CTX on the maturation, spindle morphology and aneuploidy of oocytes. **(A,B)** Rate of GVBD and PB1 extrusion of *in vitro* maturation oocytes from the two groups. **(C)** Analysis of superovulated oocyte numbers with or without CTX treatment after hCG injection. **(D)** Percentage analysis of aberrant morphology oocytes from two groups of mice. **(E)** Representative images of maturation of oocytes in **(B)**. Scale bar = 100 μm **(F)** Representative images of aberrant oocytes in **(D)**. Scale bar = 100 μm. **(G)** Typical images of correction and aberrant spindle assembly in oocytes. Scale bar = 50 μm **(H)** Statistical analysis of incorrect assembly of spindles between the two groups of mice. **(I)** Representative chromosome spread images of euploid (20 pairs) and aneuploidy (19 or 21 pairs) oocytes. Scale bar = 10 μm **(J)** Analysis of the differential proportion of aneuploidy between oocytes retrieved from the two groups. Data was presented as mean ± SEM. **P < 0.01, ***P < 0.001.

To investigate the effect of CTX on oocyte developmental competency, we isolated fully grown GV stage oocytes and performed an *in vitro* maturation experiment. We observed that the GVBD (germinal vesicle breakdown) rate was not significantly different between the two groups (93.21 ± 0.84%, *n* = 144 vs. 92.94 ± 2.40%, *n* = 123) (Figure 2A). We continued detecting meiotic progress *in vitro* and found that the percentage of PBE (polar body extrusion) was decreased after CTX exposure (92.43 ± 0.85%, *n* = 129, vs. 81.32 ± 1.78%, *n* = 113) (Figures 2B,E). However, the time of PBE was not affected, which means that CTX pre-treatment may not influence meiotic progress but maturation competency. We also superovulted mice with hCG and noticed a reduced number of MII oocytes (25.45 ± 1.34, *n* = 11 vs. 7.31 ± 0.99, *n* = 19) from CTX group mice, while the degeneration rate was higher (12.58 ± 1.11% vs. 33.87 ± 1.69%, *n* = 3) (Figures 2C,D,F). This result suggested that CTX not only decreased oocyte numbers but also impaired oocyte maturation during ovulation.

The maturation of oocytes is most related to the meiosis process, and aberrant meiotic function during oocyte maturation also impacts spindle assembly and chromosome segregation. We stained α-tubulin to observe the morphology of spindles and found that the rate of abnormal spindles from MII oocytes retrieved *in vivo* was significantly higher than that in the group without CTX injection (14.12 ± 1.40%, *n* = 93 vs. 34.64 ± 2.39%, *n* = 84) (Figures 2G,H), which revealed the impairment of oocyte nuclear maturation. Incorrect spindle assembly would also induce chromosome misalignment. We further performed chromosome spread experiments to observe the rate of faithful chromosome segregation in MII oocytes from CTX-treated mice. The percentage of aneuploidy (more or less than 20 pairs) was comparably higher than that in the control group (15.75 ± 1.53%, *n* = 96 vs. 35.20 ± 3.60%, *n* = 119) (Figures 2I,J). These results indicated the long-term toxic effect of CTX on oocytes producing correct gametes for fertilization.

### CTX Decreases Mitochondrial Function and Induces ER Stress in Oocytes

Mitochondrial function is important in maintaining oocyte quality and provides ATP during oocyte maturation and later fertilization. Alterations in the mitochondrial membrane potential (MMP or JC-1) and ROS could reflect mitochondrial indexes in oocytes. We first examined mitochondrial JC-1 staining and found that the ratio of aggregates/monomers decreased in the CTX group (1.00 ± 0.03, *n* = 48 vs. 0.70 ± 0.03, *n* = 48) (Supplementary Figures 1A,C). Moreover, ROS levels were measured by DCFH-DA staining. As shown in Supplementary Figures 1B,D, the ROS signal was significantly higher in oocytes exposed to CTX exposures. (1.00 ± 0.04, *n* = 53 vs. 1.63 ± 0.05, *n* = 53) Mito-Tracker staining was used to assess the activity and distribution of mitochondria. Perinenuclear accumulation and homogeneity of mitochondria were observed in the majority of normal oocytes, and the proportion of clustered mitochondria was higher in the CTX group (13.35 ± 1.24, *n* = 60 vs. 27.61 ± 2.15, *n* = 58) (Supplementary Figures 1E,F). We also measured the ATP content of mature oocytes and found that it was lower in CTX-treated oocytes than in normal oocytes (1.00 ± 0.02, *n* = 35 vs. 0.84 ± 0.02, *n* = 35) (Supplementary Figure 1G), which was consistent with the fluorescence staining detection used above. These results suggested that early maternal exposure to CTX damages mitochondrial function and impairs oocyte quality.

The mitochondria-associated ER membrane is also important for maintaining normal cellular function. ER stress occurs when the protein-folding capacity of the ER is negatively affected, and GRP78 is a sensor protein. We measured the protein expression of GRP78 in oocytes and found that the signal was comparably higher in the CTX group (1.00 ± 0.08, *n* = 22 vs. 1.63 ± 0.12, *n* = 26) (Supplementary Figures 2A,B). Moreover, alterations in the mRNA expression of GRP78, ATF4 and CHOP also showed the same results (Supplementary Figure 2C). We next examined the distribution of ER by ER-tracker. As shown in Supplementary Figure 2B, in the normal group, ER was mainly distributed around the spindle but homogenous in post CTX-treated oocytes (15.61 ± 2.35%, *n* = 66 vs. 44.16 ± 4.70%, *n* = 64) (Supplementary Figure 2E). These results demonstrated that ER distribution was also affected and that CTX caused ER stress in oocytes even post treatment for almost 2 months.

### Detrimental Effects of CTX on Embryo Developmental Competence

**FIGURE 3:**
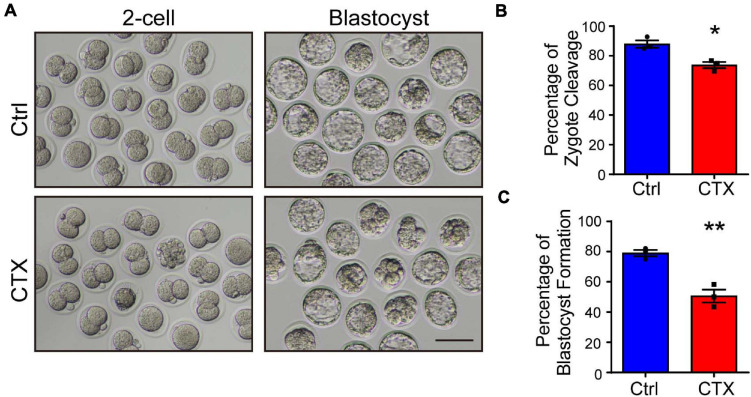
Detrimental effects of CTX on embryo developmental competence. **(A)** Representative images of the developmental stage of 2-cell and blastocyst formation after *in vitro* fertilization of oocytes with or without CTX exposure. **(B,C)** The percentage of IVF embryo development rates between the two groups. Scale bar = 100 μm. Data was expressed as mean ± SEM. * *P* < 0.05, **P < 0.01.

Oocyte defects directly impairs further fertilization and embryonic development. Misaligned chromosomes cause aneuploidy, which will induce embryo arrest during division. We performed an IVF experiment and analyzed the percentage of cleavage and blastocyst formation. Embryos from control group mice showed normal development, but the 2-cell and blastocyst rates were significantly lower (2-cell, 87.99 ± 2.46%, *n* = 104 vs. 73.75 ± 2.14%, *n* = 77, blastocyst, 79.05 ± 2.12% vs. 50.60 ± 4.30%, *n* = 77) (Figures 3A–C). These data demonstrated that maternal exposure to CTX earlier would also have detrimental effects on embryo developmental competence whether *in vitro*-assisted reproduction techniques were utilized.

### CTX Alters Histone Modification and DNA Methylation and/or Demethylation Levels in Oocytes

**FIGURE 4:**
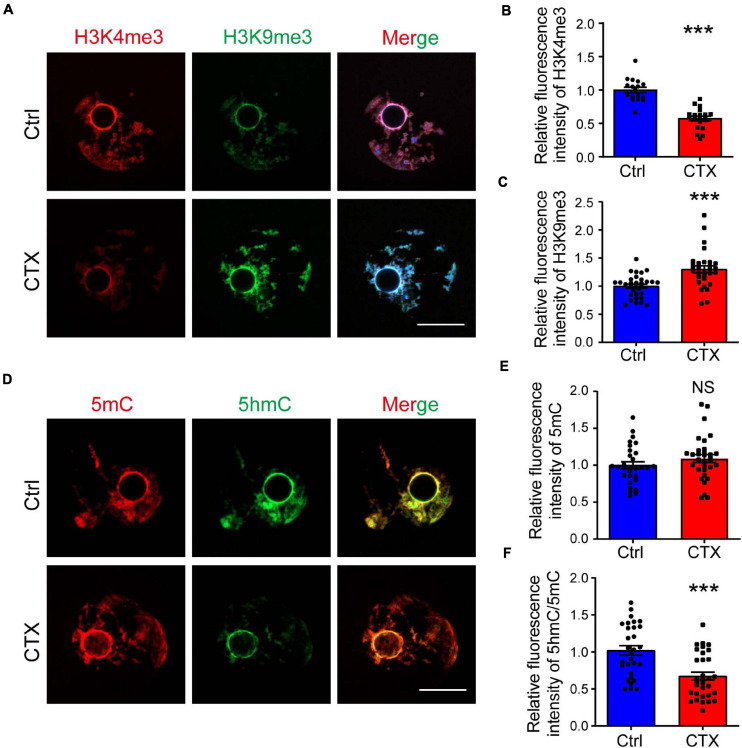
CTX affects histone modification and DNA methylation levels in fully grown GV oocytes. **(A)** Immunostaining of H3K4me3 and H3K9me3 in fully grown GV oocytes after CTX exposure. Red fluorescence represents H3K4me3, while green fluorescence represents H3K9me3. **(B,C)** Measurement of increased levels of relative fluorescence intensity of H3K4me3 and decreased levels of H3K9me3 in post-CTX oocytes. **(D,E)** Immunofluorescence staining of 5′-methylcytosine (5mC) and 5′-hydroxymethylcytosine (5hmC) in oocytes. **(F)** Statistical analysis of the 5mC level showed no significant difference but a decrease in the 5hmC/5mC ratio. Scale bar = 50 μm. The intensity was measured by Image J software and data was presented as mean ± SEM. NS, no significant, ***P < 0.001.

The low developmental capacity of oocytes and embryos is also related to histone modification alterations. We analyzed the histone methylation of fully grown oocytes and showed that the fluorescence intensity of H3K4me3 was higher in the group without CTX exposure (1.00 ± 0.04, *n* = 17 vs. 0.57 ± 0.04, *n* = 20) (Figures 4A,B). However, the change in H3K9me3 was exactly the opposite, and the intensity level of oocytes after CTX treatment was higher (1.00 ± 0.04, *n* = 32 vs. 1.30 ± 0.06, *n* = 30) (Figures 4A,C). Methylation of the 5-position of cytosines is also an important component to maintain DNA methylation during oocyte maturation (Iqbal et al., 2011). We found that the level of 5mC immunostaining was not significantly different (1.00 ± 0.047, *n* = 30 vs. 1.09 ± 0.05, *n* = 32) (Figures 4D,E). However, the level of 5hmC vs. 5mC was downregulated (1.00 ± 0.06, *n* = 30 vs. 0.67 ± 0.05, *n* = 32) (Figures 4D,F), which is crucial for embryo development and indicated that DNA demethylation capacity was impaired. These results demonstrated that epigenetic regulation was affected in the long term after CTX treatment and rendered the developmental competency of oocytes.

### CTX Treatment Exposure in Primordial Follicle Represses Maternal Factors’ Transcriptional Accumulation in Fully Grown GV Oocytes

**FIGURE 5:**
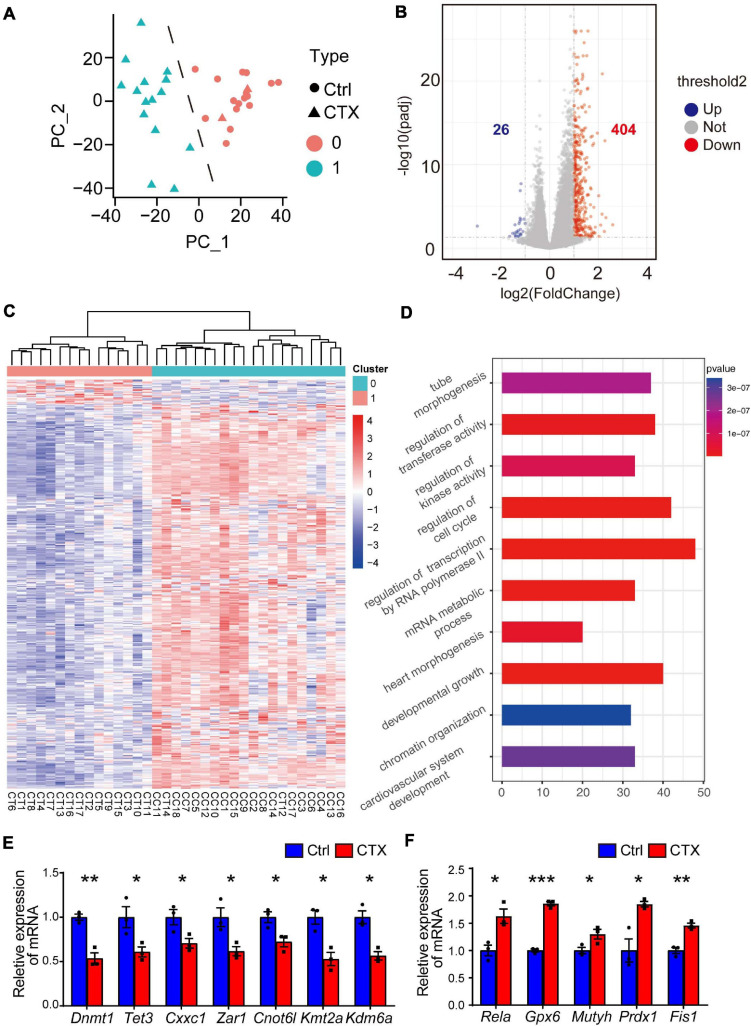
Single-cell RNA transcriptional profiling of oocytes within/without CTX early maternal exposure. **(A)** Principal component analysis (PCA) exhibiting the distinguishing clusters of samples from two groups. **(B)** Volcano plots with differentially expressed genes are shown. (*p* < 0.05 and log2-fold change > 1). **(C)** Heatmap of all the up- and down-regulated genes with the most significant fold change between the two clusters. There were 2 samples from the CTX group divided into Cluster 0. **(D)** GO analysis of highly expressed genes enriched in Cluster, which is downregulated in CTX-exposed oocytes. **(E–F)** RT-qPCR analysis of some typical down- and up-regulated genes in single-cell RNA-seq. Data was presented as mean ± SEM. *P < 0.05, **P < 0.01, ***P < 0.001.

To further investigate the potential mechanism for the decreased capacity of oocyte maturation and development, we performed single-cell RNA-seq analysis to detect the differentially expressed genes of GV oocytes that developed from primordial follicles that survived chemotherapy with CTX. We collected a total of 35 oocytes (18 from the control group and 17 from the CTX group) and tested the correlations among all the samples. The single cell sequencing quality control (QC) is shown in Supplementary Table 3, and oocytes after CTX treatment have comparable qualities as control oocytes. Principal component analysis (PCA) analysis showed that samples were divided into two distinct clusters, showing the difference among oocytes retrieved from different groups of mice. Interestingly, we found 2 samples from CTX-treated oocytes in Cluster 0, including 18 oocytes from control group and 2 oocytes from CTX group, which may with better capacity and quality of development, was defined as high quality oocytes, while Cluster 1 was defined as low quality oocytes (Figure 5A). These results suggested that a proportion of oocytes developed as normal capacity after chemotherapy. We then analyzed the general gene expression between two clusters of oocytes (Figure 5B). Among this, 26 genes were upregulated and 404 genes expression was decreased from Cluster 1 compared to Cluster 0. Moreover, based on fold change, a heatmap of enriched genes all the differentially up- and down-expressed genes between the two clusters of oocytes (Figure 5C). As shown in Figure 5D, Gene Ontology (GO) analysis showed that multiple biological processes, specifically the regulatory regions of transcription, RNA polymerase II and chromatin, were affected. We also verified some genes that will affect the meiosis process and early embryo development, and the expression of these genes was quantified by real-time qPCR, which was consistent with the single-cell RNA-seq results (Figure 5E). We also found that some peroxidase and glutathione transferase activity-related genes were expressed at higher levels after CTX exposure, such as *Prdx1* and *Fis1*, which are involved in modulating oxidative stress (Figure 5F). These results suggest CTX pre-pubertal exposure would impair oocyte quality through affecting transcriptional activity.

### Role of YAP1 in Embryo Developmental Competence After CTX Exposure

**FIGURE 6:**
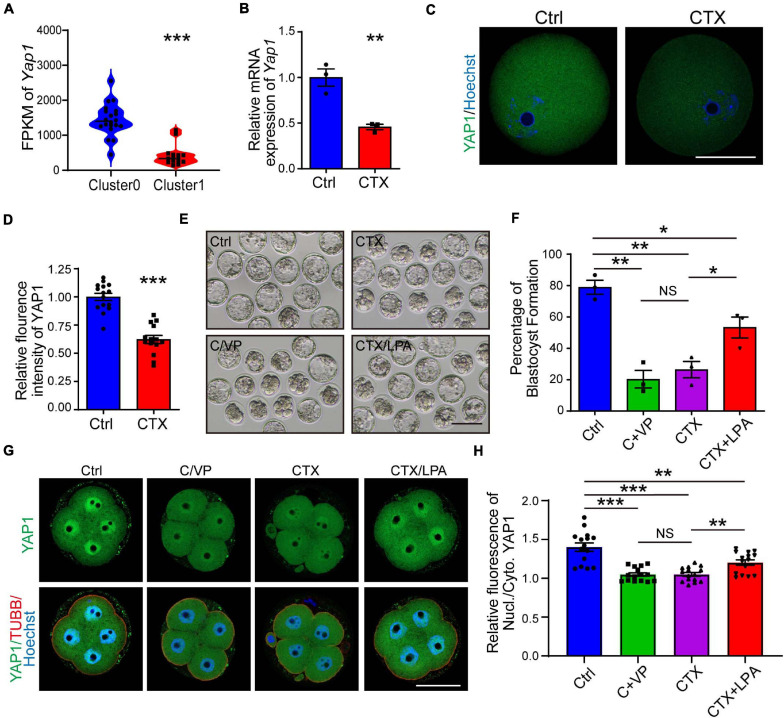
Expression of YAP1 and its role in embryonic development in CTX-treated oocytes. **(A)** FPKM level of YAP1 expression from single-cell sequencing data between two clusters of oocytes. **(B)** The relative mRNA expression level of *Yap1* in oocytes among groups. **(C)** Immunofluorescence of YAP1 in GV oocytes collected from mice in the CTX group. Scale bar = 50 μm. **(D)** Relative fluorescence intensity analysis of YAP1 in oocytes. **(E)** Representative images of blastocyst formation in the four culture groups. Ctrl, Control; C/VP, control + VP; CTX, cyclophosphamide; LPA, lysophosphatidic acid. Scale bar = 100 μm. **(F)** Comparable analysis of the four embryo culture compositions in **(D)**. Data was expressed as mean ± SEM. **(G)** Localization of YAP1 in four-cell embryos in four groups of **(D)**. **(H)** Relative immunofluorescence of YAP1 signals in the nucleus versus in the cytoplasm. TUBB, beta-tubulin, Nucl, nucleus; Cyto, cytoplasm. Scale bar = 50 μm. 50, *P < 0.05, **P < 0.01, ***P < 0.001.

YAP1, a key maternal factor to facilitate zygotic genome activation, is significantly decreased observed in the RNA-seq results, based on Fragments Per Kilobase per Million (FPKM) (1443 ± 102.50, *n* = 18, vs. 423.5 ± 75.04) (Figure 6A). First, we verified the mRNA and protein expression (1.00 ± 0.03, *n* = 15 vs. 0.62 ± 0.04, *n* = 14) of oocytes, and both were downregulated in the CTX group, which was correlated with the single-cell RNA-seq results (Figures 6A–D). To mimic the phonotype of YAP1 decreased embryonic development, we cultured normal group zygote in presence of 0.5μM verteporfin (VP), which is known to inhibit YAP–TEAD4 target genes. VP addition decreased further embryo development (Ctrl 79.45 ± 2.23%, *n* = 111 vs. VP 50.13 ± 2.77%, *n* = 110) (Figures 6E,F). Moreover, we used media with 10 μM LPA to rescue the low competency of CTX oocytes. As shown in Figure 6E, LPA partially improved the formation of blastocysts from CTX-treated oocytes (Ctrl 79.45 ± 2.23%, *n* = 111 vs. CTX 53.60 ± 2.60%, *n* = 81 vs. CTX + LPA 66.65 ± 3.33%, *n* = 75) (Figure 6F). Furthermore, we collected 4-cell stage embryos from these four groups and detected the sub-cellular localization. Embryos from control group showed more intensive nucleus intensity versus cytoplasm while there was no significant localization difference in nucleus or cytoplasm of CTX group embryos (Figure 6G). Verteporfin treated reduced the nuclear intensity of YAP1, but LPA could activate and prompt the YAP1 nuclear localization (Ctrl 1.40 ± 0.21, *n* = 15 vs. VP 1.04 ± 0.08, *n* = 15, CTX 1.05 ± 0.09, *n* = 15 vs. CTX + LPA 1.20 ± 0.14, *n* = 15) (Figure 6H). The sub-localization of YAP1 alteration among four groups was consistent with the formation ratio of blastocyst. These results suggested the role of YAP1 in the developmental competence of preimplantation embryos with CTX exposure.

## Discussion

**FIGURE 7:**
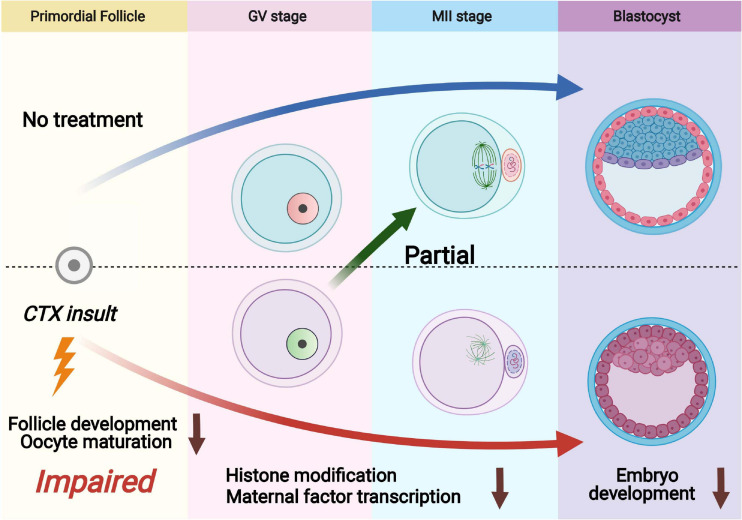
Schematic diagram showing dysfunction of oocytes surviving from primordial follicles with pre-pubertal exposure to cyclophosphamide. Cyclophosphamide (CTX) causes overactivation of primordial follicles and induces DNA damage and eventually apoptosis as an acute toxic effect. A number of primordial follicles can survive chemotherapy and develop to the antral stage. The oocytes that survived CTX insult exhibited upregulation of H3K9me3, reduced H3K4me3 and low levels of 5hmC/5mC. Meanwhile, the maternal mRNAs were insufficiently accumulated after CTX insult, leading to low quality of mature oocyte (aneuploidy and mitochondrial dysfunction) and eventually affecting early embryo development.

Cyclophosphamide (CTX) is a widely used chemotherapeutic and immunosuppressive agent to treat a range of tumors and other autoimmune diseases. Although the fertility risk of CTX is well known after chemotherapy, the lasting effect of CTX in oocytes is still not clearly demonstrated (Cui et al., 2018; Hao et al., 2019). Several studies have reported that CTX exposure also has the ability to give birth in human and animal models (Di Emidio et al., 2019). However, whether the surviving primordial follicles that can escape and be repaired from CTX damage still have the capacity of maturation and developmental competency is not clarified. In this study, we investigated the long-term detrimental effects of cyclophosphamide on follicles that survived treatment with the anticancer agent CTX and found wide-ranging deleterious effects after early maternal exposure to CTX with decreased oocyte quality (Figure 7).

The follicle serves as a basic component in the ovary, and the number of follicles is determined at birth, which is also defined as fertility preserve (Zhang and Liu, 2015). Throughout the reproductive life of females, primordial follicles remain silent and activate in a balance (Reddy et al., 2008). Our study demonstrated that CTX affected mouse ovarian weight and that the number of oocytes in the ovary was also reduced. Cyclophosphamide has long-term adverse effects on follicular growth and angiogenesis, as previous research reported, which suggests that ovary microenvironment impairment leads to follicle development defects (Ezoe et al., 2014). Furthermore, we found that the maturation capacity of the oocyte was also reduced with a decreased rate of polar body extrusion. Interestingly, we did not observe comparable times of GVBD and PBE, and CTX may impair but not prolong meiotic progression. Research in the 1990s confirmed that embryo cleavage was reduced after prime-CTX administration, but a recent study revealed that early development was not influenced (Pydyn and Ataya, 1991; Salian et al., 2020). In our study, the results showed that both early cleavage and later blastocyst formation declined. The reason for these differences may partly be the use of distinguished dosages and manufacturer-derived agents (Gonfloni et al., 2009; Kerr et al., 2012). We also observed spindle assembly and chromosome segregation defects in oocytes from the CTX-administered group, and these aberrant phenotypes are considered crucial for increasing infertility incidence (Barekati et al., 2008; Li et al., 2013). Meanwhile, an earlier study found that exposure to CTX *in vivo* had adverse effects on oocyte function, and it can be partially reversible. However, pregnancy failure may also be related to uterus- and pregnancy-supporting dysfunction (Garg et al., 2020; Griffiths et al., 2020).

Mitochondria are organelles that play a vital role in complex processes, including metabolism, signaling and programmed cell death (Li et al., 2020). During oocyte maturation and fertilization and early embryo development, mitochondria provide energy through oxidative phosphorylation, which is critical for transcription and translation. Mitochondrial dysfunction leads to increased reactive oxygen species production and reduced ATP synthesis, which are involved in decreased oocyte quality. mRNA transcriptional results suggested the upregulation of *Rela*, *Gpx6*, and *Fis1*, which are involved in critical roles in mitochondrial-mediated apoptosis and the accumulation of ROS (Khandelwal et al., 2011; Tadros et al., 2014; Xu et al., 2015; Joshi et al., 2018). In our results, we detected a decrease in mitochondrial membrane potential and ATP content, accompanied by aberrant mitochondrial distribution. In oocytes, the balance between the production and scavenging of ROS is important to oocyte homeostasis and quality (Jeelani et al., 2017), and an *in vitro* study reported that CTX could induce ROS in cultured oocytes and accelerate oocyte aging (Goud et al., 2008). Excess ROS leads to increased DNA damage and apoptotic cascade activation, which affect oocyte survival in CTX-exposed females. PRDX1 is the component of antioxidant systems that prevents ROS-mediated inhibition of telomerase (Ahmed and Lingner, 2018). 8-Oxoguanine (8-oxoG) is a prevalent genotoxic lesion that is generated in DNA attacked by ROS. Moreover, MUTYH is a DNA repair enzyme that recognizes and removes 8-oxoG and initiates base excision repair (Lu et al., 2015; Chen et al., 2019). Increasing the expression of *Prdx1* and *Mutyh* may be involved in the response to reactive oxidative stress, and we found that these genes were expressed at higher levels after nearly 2 months of CTX injection. It is possible that their upregulated expression is a compensation mechanism for oxidative stress and maintains the hemostasis of low-quality oocytes for long-term development. Clinical research has also revealed that melatonin supplementation promotes the development of immature human oocytes into healthy offspring by protecting mitochondrial function, which suggests that reducing the level of ROS may be important in revising low-quality oocytes (Guo et al., 2020). In addition, methylation may also interfere with oxidative stress, inducing metabolic changes in glutathione synthesis and homocysteine (Liu et al., 2017). The ER is not only spatially but also functionally related to mitochondria. We examined the distribution and function of ER and showed that the ER distribution was affected after CTX exposure. Excessive unfolded proteins in the ER lumen accumulate and cause ER stress, leading to the activation of the unfolded protein response (UPR) (Shore et al., 2011). ER stress and UPR activation are involved in the development and pathogenesis of various diseases, especially genetic disorders and metabolic dysfunction (Jin et al., 2020). In BM-resident lymphoma cells, CTX induced ER stress that resulted in the ATF4-mediated pathway. Here, we detected *Grp78*, *Atf4*, and *Chop*, which are considered markers of ER stress, and showed that their expression was increasingly affected after CTX exposure and that ER stress was induced. Deficient of mitochondrial and endoplasmic reticulum contributed important roles in oocyte decline after CTX treatment prepubertally.

Previously, CTX-treated mice were reported to have a significant reduction in primordial and growing follicles, which is associated with follicle activation and apoptosis. It can be prevented by the administration of AS101, a ferto protector (Di Emidio et al., 2017). However, a recent study showed that even though AS101 could maintain follicle reserve, it could not totally protect the methylation level of oocytes (Di Emidio et al., 2019). Epigenetic modifications are involved in changes in DNA and genome stability (Jeelani et al., 2017). Histone modifications are an important component of epigenetic reprogramming and will accomplish later developmental events. In our results, the trimethylation level of H3K4 was decreased, while H3K9 was upregulated. H3K4me3 is crucial for the differentiation of embryonic stem cells and regulates the developmental process during oogenesis and embryogenesis (Zhang et al., 2016). CXXC finger protein-1 (CFP1) is encoded by the *Cxxc1* gene in mice, and maternal deficiency of CFP1 leads to decreased maturation and developmental competence of oocytes, which is mediated by H3K4me3 in developing oocytes (Yu et al., 2017). Meiotic progression was also affected by the CFP1 regulatory H3K4me3 mechanism in mouse oocytes (Sha et al., 2018a). We found that Cxxc1 gene expression was significantly downregulated after CTX exposure, which may explain the loss capacity of oocytes in our mouse model. H3K9me3 is associated with heterochromatin and gene repression and acts as a barrier to cell fate changes, which must be reprogrammed after fertilization (Becker et al., 2016; Wang et al., 2018). KDM4A-mediated H3K9me3 demethylation in oocytes is crucial for normal preimplantation development. H3K9me3 demethylase Kdm4a deficiency caused embryo arrest in mice, and Kdm4d overexpression improved the blastocyst development of transplanted SCNT embryos of monkeys, suggesting important roles of H3K9me3 demethylation in oocytes and embryos (Liu et al., 2018; Sankar et al., 2020). In Stella-deficient mice, DNMT1 plays a critical role in generating the aberrant DNA methylome in oocytes (Li et al., 2018). Our RNA-seq data suggested that *Dnmt1* decreased severely after CTX exposure, which may decrease the capacity to prevent aberrant DNA methylation. Although in our results, we found that DNA methylation establishment was not significantly affected, the balance of demethylation may be impaired. Maternal TET3 could convert 5mC to 5hmC during oocyte maturation and fertilization for DNA demethylation, which is crucial for the developmental competence of embryos (Gu et al., 2011). Our results showed defects in the 5mC to 5hmC transition, which will be a potential mechanism of loss capacity during further embryo development. In a polycystic ovarian syndrome PCOS-like mouse model, methyl donor dietary supplementation normalized aberrant genomic DNA levels of methylation (Mimouni et al., 2021). Identifying molecular compounds that can alter methylation levels may protect against CTX-induced persistent genomic integrity deficiency.

During the early development of embryos, maternal-zygotic transition is important to correctly control later embryo development (Schier, 2007; Sun et al., 2007). YAP1 is a transcriptional co-activator of TEAD family transcription factors that is essential for zygotic genome activation (ZGA), and maternal knockout of *Yap1* prolongs and impairs embryo development (Yu et al., 2016a). LPA addition promoted YAP1 nuclear localization and upregulated downstream gene expression. *Yap1* was involved in the enrichment of differentially expressed genes with high expression levels. To rescue embryo development-deficient *in vitro* incubation, we added LPA to the culture media to prompt YAP1 nucleus localization and improve the competency of blastocyst formation. However, the addition of LPA partially increased the rate of blastocysts during embryo development. It may be that CTX administration downregulated a number of maternal factors, such as *Cnot6l* and *Zar1*, but not only *Yap1*, which has a negative impact on oocytes and embryos (Sha et al., 2018b; Rong et al., 2019). Activation of YAP signaling cannot completely recuse other gene dysfunction in the developmental capacity of embryos.

In summary, animal models have confirmed that short-term exposure to CTX will have a long-term lasting effect. These results suggest that IVF and embryo cryopreservation of patients suffering from chemotherapy should be more cautious and evaluated (Meirow et al., 2001). Even though acute DNA damage has already been repaired, long-term genomic toxicity still exists. Follicle development, oocyte maturation and embryo development are both impaired. Oocyte quality declines following mitochondrial dysfunction and ER dysfunction, which should attract more attention. Activation of YAP1 could partially improve embryo development while using assisted reproductive technology. Further detection of important targets by using advanced sequencing techniques may provide a new conception of oncofertility and call a new approach to fertility preservation.

## Data Availability Statement

The data presented in the study are deposited in the NCBI database, accession number (PRJNA725889; https://www.ncbi.nlm.nih.gov/bioproject/PRJNA725889).

## Ethics Statement

The animal study was reviewed and approved by the Institutional Animal Care and Use Committee (IACUC) of Zhejiang University.

## Author Contributions

SZ and YLZ conceptualized the study. WY and YLZ led the experimental design and wrote the experimental design. WY, YM, and JJ performed the experiments. WY and YM analyzed the data. WY and YLZ wrote the manuscript. All other authors were involved in analyzing and interpreting the data and corrected the manuscript.

## Conflict of Interest

The authors declare that the research was conducted in the absence of any commercial or financial relationships that could be construed as a potential conflict of interest.

## Supplementary Material

Supplementary Figure 1 Dysfunction of mitochondria after long-term insult toxic effects. **(A)** Typical picture of JC-1 red channels and green channels in oocytes between the two groups. Red, JC-1 Aggregates; Green, JC-1 Monomer. **(B)** Representative oocyte ROS levels with DCHF-DA fluorescence (green). **(C,D)** Relative intensity fluorescence analysis of JC-1 and ROS staining in **(A,B)**. **(E)** Typical picture of mitochondrial distribution labeled with MitoTracker. **(F)** Proportion of abnormally localized mitochondria after CTX treatment among the groups. **(G)** The difference of oocyte ATP content. Scale bar = 50 μm. Data was presented as mean ± SEM. ^∗∗^*P* < 0.01, ^∗∗∗^*P* < 0.001.

Supplementary Figure 2 Effects of CTX on ER function in oocytes with CTX exposure. **(A)** Immunostaining of GRP78 protein in MII oocytes; GRP78 expression was higher after CTX treatment. **(B)** Relative intensity analysis of GRP78 fluorescence. **(C)** qPCR reveals the relative mRNA expression of molecular markers of ER stress, including *Grp78*, *Atf4*, and *Chop*. **(D)** Representative image of ER distribution of normal and post-CTX-treated oocytes. **(E)** Statistical analysis of the rate of abnormal ER distribution. Scale bar = 50 μm. Data was presented as mean ± SEM. ^∗^*P* < 0.05, ^∗∗^*P* < 0.01, ^∗∗∗^*P* < 0.001.
