# Supplementary figures and images for: Cyclophosphamide Exposure Causes Long-Term Detrimental Effect of Oocytes Developmental Competence Through Affecting the Epigenetic Modification and Maternal Factors’ Transcription During Oocyte Growth
Source: Front Cell Dev Biol. 2021 Jun 7;9:682060. doi: 10.3389/fcell.2021.682060 (PMC8215553; doi:10.3389/fcell.2021.682060)

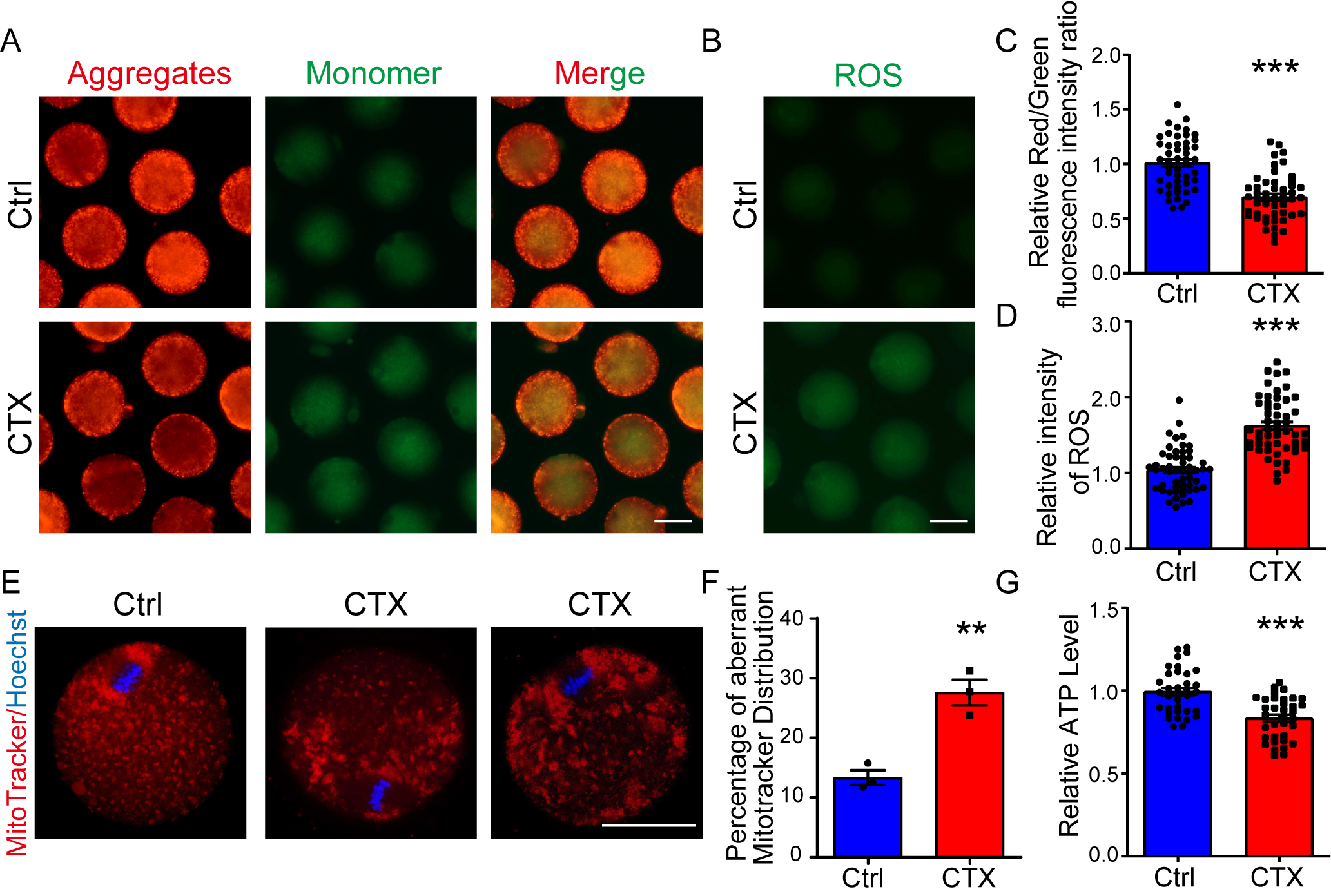

Supplement: Supplementary Figure 1 — Dysfunction of mitochondria after long-term insult toxic effects. (A) Typical picture of JC-1 red channels and green channels in oocytes between the two groups. Red, JC-1 Aggregates; Green, JC-1 Monomer. (B) Representative oocyte ROS levels with DCHF-DA fluorescence (green). (C,D) Relative intensity fluorescence analysis of JC-1 and ROS staining in (A,B). (E) Typical picture of mitochondrial distribution labeled with MitoTracker. (F) Proportion of abnormally localized mitochondria after CTX treatment among the groups. (G) The difference of oocyte ATP content. Scale bar = 50 μm. Data was presented as mean ± SEM. ∗∗P < 0.01, ∗∗∗P < 0.001. [file Image_1.tif]

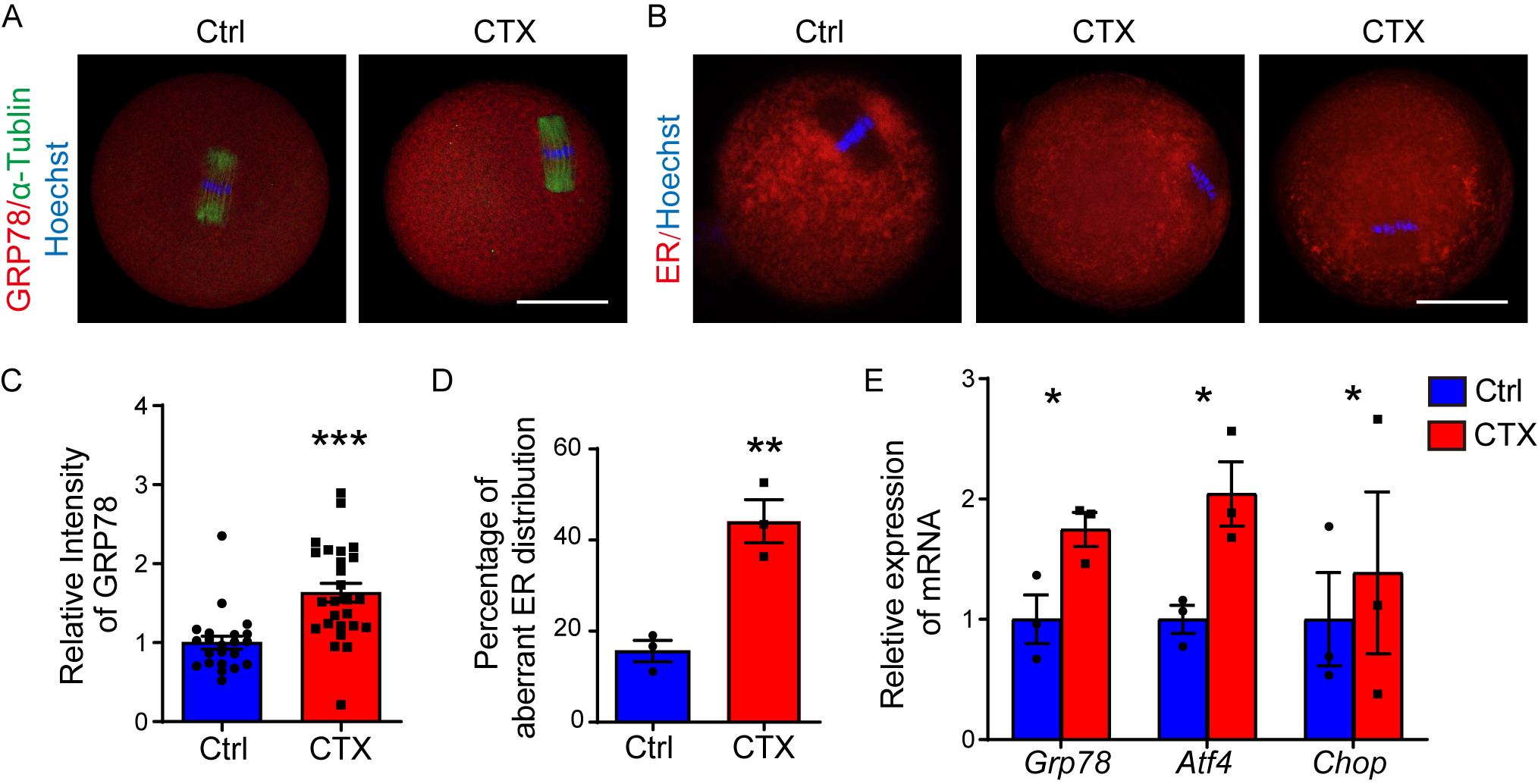

Supplement: Supplementary Figure 2 — Effects of CTX on ER function in oocytes with CTX exposure. (A) Immunostaining of GRP78 protein in MII oocytes; GRP78 expression was higher after CTX treatment. (B) Relative intensity analysis of GRP78 fluorescence. (C) qPCR reveals the relative mRNA expression of molecular markers of ER stress, including Grp78, Atf4, and Chop. (D) Representative image of ER distribution of normal and post-CTX-treated oocytes. (E) Statistical analysis of the rate of abnormal ER distribution. Scale bar = 50 μm. Data was presented as mean ± SEM. ∗P < 0.05, ∗∗P < 0.01, ∗∗∗P < 0.001. [file Image_2.tif]
