# Supplementary material for: Cyclophosphamide Exposure Causes Long-Term Detrimental Effect of Oocytes Developmental Competence Through Affecting the Epigenetic Modification and Maternal Factors’ Transcription During Oocyte Growth
Source: Front Cell Dev Biol. 2021 Jun 7;9:682060. doi: 10.3389/fcell.2021.682060 (PMC8215553; doi:10.3389/fcell.2021.682060)
Supplement: Supplementary file 6 [file Table_4.docx]

Supplementary Table S4 Real-time qPCR Primers

| Gene | Forward primer | Reverse primer |
| --- | --- | --- |
| *Actin* | 5’-CCGTAAAGACCTCTATGCC-3′ | 5′-CTCAGTAACAGTCCGCCTA-3′ |
| *Dnmt1* | 5′-AAGAATGGTGTTGTCTACCGAC-3′ | 5′-CATCCAGGTTGCTCCCCTTG-3′ |
| *Tet3* | 5′-TGCGATTGTGTCGAACAAATAGT-3′ | 5′-TCCATACCGATCCTCCATGAG-3′ |
| *Cxxc1* | 5′-TTGGATGTGACAACTGCAACG-3′ | 5′-GTGGCGGTAACGAATCTCCAG-3′ |
| *Zar1* | 5′-TCGGTGCAGTGTTCACTCG-3′ | 5′-CTACGGTCTGCCAGGATCG-3′ |
| *Cnot6l* | 5′-AAAATCTCACTGGGCAGAGTTAG-3′ | 5′-TGCGAGCAAGGTTATTGTCATT-3′ |
| *Kmt2a* | 5′-GCAGATTGTAAGACGGCGAG-3′ | 5′-GAGAGGGGGTGTTCCTTCCTT-3′ |
| *Kdm6a* | 5′-CGGGCGGACAAAAGAAGAAC-3′ | 5′-CATAGACTTGCATCAGATCCTCC-3′ |
| *Rela* | 5′-AGGCTTCTGGGCCTTATGTG-3′ | 5′-TGCTTCTCTCGCCAGGAATAC-3′ |
| *Gpx6* | 5′-GCCCAGAAGTTGTGGGGTTC-3′ | 5′-TCCATACTCATAGACGGTGCC-3′ |
| *Mutyh* | 5′-AGGTTGCCACAGTGATCGAC-3′ | 5′-TAGCTCCTTCTTGTAGCCGAC-3′ |
| *Prdx1* | 5′-AATGCAAAAATTGGGTATCCTGC-3′ | 5′-CGTGGGACACACAAAAGTAAAGT-3′ |
| *Fis1* | 5′-TGTCCAAGAGCACGCAATTTG-3′ | 5′-CCTCGCACATACTTTAGAGCCTT-3′ |
